# Special Issue “Theoretical Calculation and Molecular Modeling of Nanomaterials”

**DOI:** 10.3390/nano13172473

**Published:** 2023-09-01

**Authors:** Frederik Tielens

**Affiliations:** General Chemistry (ALGC)—Materials Modelling Group, Vrije Universiteit Brussel, Pleinlaan 2, 1050 Brussel, Belgium; frederik.tielens@vub.be

The continuous advancement of computational chemistry and the chemical modeling of materials is closely aligned with the ever-evolving computational power and related techniques. This ongoing evolution has placed chemists’ skills at the forefront. Regardless of whether Moore’s law is adhered to, the focus has shifted towards creating the models themselves. While there is room for improvement in calculation accuracy, the fundamental chemical properties and trends are now relatively well replicated. Density functional theory (DFT) can be regarded as a mature technique, serving as a reliable predictive tool in material science applications. Classical molecular dynamics remains indispensable for studying large systems, but emerging methods like AI-based approaches are also gaining prominence. We are entering an exciting era where our own creativity becomes the only limit to understanding our environment—a time of multiscale modeling. This Special Issue aims to emphasize the development of relevant models that accurately describe and predict the chemistry of nanomaterials, within the constraints of available computational resources and techniques. In summary, the diverse applications of these models have generated immense research interest in the field of material modeling. This Special Issue aims to cover a broad spectrum of these applications. We hope that the contributions to this second Special Issue on the topic [1] provide readers with valuable insights into recent advances in this field, while also offering guidance for the future development of materials. I extend my gratitude to all the authors who have made this Special Issue possible.

In this Special Issue, we have the following contributions that contribute to the richness of variety in the field of materials modelling.

In this first study [2], the properties of transition metal phthalocyaninato complexes (MPc) were investigated to understand the nature of M–Pc bonding. Theoretical calculations revealed that this bonding is primarily dominated by σ interactions, with FePc exhibiting the strongest and most covalent M–Pc bond. Additionally, spectroscopic analysis provided insights into the unoccupied electronic structure of the MPc complexes, highlighting the presence of specific electronic states and transitions involving metal–ligand charge transfer and the lowest-lying π* orbital of the phthalocyaninato ligand.

In the second study [3], the interactions between the hydroxyurea (HU) drug and pristine C60 and heterofullerenes MC59 (M = B, Si, Al) were investigated using density functional theory calculations. The results indicate that HU molecule chemisorbs onto BC59, SiC59, and AlC59 with moderate adsorption energy and noticeable charge transfer, suggesting the potential of these heterofullerenes as carriers for hydroxyurea drug delivery.

Next, the effect of aluminum doping on the surface morphology of ultra-thin silver films was investigated using molecular dynamics simulations. The addition of aluminum resulted in reduced surface roughness compared to pure silver films, and the smoother surface was attributed to the immobilization of silver atoms on the substrate due to the anchoring effect of aluminum dopants. These findings suggest that aluminum doping represents a promising approach to improve the surface morphology and roughness control of ultra-thin silver films for applications such as transparent conducting electrodes [4]. 

Density functional theory (DFT) calculations were used to estimate the exfoliation energy (Eexf) of 486 different MAX phases, which are precursors to MXenes. The results show that Eexf decreases when the MAX phase is a nitride, when the metal component belongs to a d-series, when the p-block A element group is lower, and when the MXene is thicker. Additionally, Eexf was found to influence the surface chemical activity, specifically the strength of CO_2_ adsorption, with more unstable MXenes exhibiting stronger attachment of species [5].

Large-scale atomistic simulations were conducted to investigate the electrochemical and mechanical behavior of carbon-coated silicon nanotube (SiNT@CNT) anodes in lithium-ion batteries (LIBs). The simulations demonstrated that the SiNT@CNT anode exhibited higher lithium capacity and faster lithiation rate compared to its silicon nanowire (SiNW) counterparts due to the alleviation of compressive stress concentration within the SiNT structure. This study also highlighted the influence of contact mode on stress distribution and structural stability, providing insights for optimizing the charging strategies and nanostructural design of SiNT-based electrode materials [6].

Another study investigates the binding affinity of various metallic cations to phosphate and oxalate, as well as their compatibility with different anionic species for administration. Quantum chemical calculations are used to understand the competition between complexes and to propose potential improved binders for phosphate and oxalate in the treatment of hyperphosphatemia and hyperoxaluria [7].

In another paper, the impact of surface geometry on surface energy at micro/nano scales is studied using the Lennard–Jones potential, and the homogenization hypothesis is also included. The surface energy is expressed as a function of local curvatures, and its accuracy is confirmed through comparisons with experimental and numerical results. The findings reveal that surface energy decreases on convex surfaces, increases on concave surfaces, and the effect of curvatures becomes significant at scales smaller than 5 nm. This curvature-based surface energy approach provides valuable insights for designing micro/nano systems [8].

In the next study, thermally assisted occupation density functional theory (TAO-DFT) is used to accurately predict the electronic properties of carbon nanobelts (C-Belt[n]) with different sizes. The results reveal that larger C-Belt[n] exhibit a more pronounced multi-reference character, as indicated by the symmetrized von Neumann entropy and occupation numbers of active TAO orbitals, with the active orbitals being delocalized along the circumference of the nanobelts [9].

An investigation on the dynamic behaviors of aniline cations (ANI+) in their intercalation into graphite interlayers using experimental and multiscale simulation approaches is presented. Through in situ intercalation polymerization and ultrasonication, ANI+ are successfully intercalated into the graphite layers by weakening the π–π interactions and improving the intercalation ability of dissociated cations. This research provides insights into the intercalation behaviors of ANI+ and opens avenues for further exploration of organic-molecule-intercalated graphite compounds [10].

Another study explores the impact of various substitutional nitrogen doping configurations on the electrical conductivity of nitrogen-doped carbon nanotubes (N-CNTs) using hybrid density functional theory and semiclassical Boltzmann transport theory. The results demonstrate significant variations in electrical conductivity and relative energies among different dopant configurations, providing insights that can aid in optimizing the electrical transport properties of N-CNTs [11].

A work employing grand canonical Monte Carlo simulations to investigate the adsorption behavior of methane, hydrogen, and their mixture in graphene pores of different sizes is also included. This study reveals that an interlayer distance approximately twice the van der Waals distance of the adsorbate enhances the adsorbing ability, and slit-shaped graphene pores demonstrate high adsorption capacity for methane and effective separation from hydrogen in a mixture at practical working conditions [12].

The next article presents a theoretical model for a type I Weyl semimetal in the presence of torsional dislocations. Through mathematical analysis and the Kubo formalism, the effects of multiple scattering events with randomly distributed dislocations are incorporated to calculate the electronic conductivity as a function of temperature and dislocation concentration. Analytical formulas are applied to predict the electrical conductivity of transition metal monopnictides, including TaAs, TaP, NbAs, and NbP [13].

Another work presents the synthesis and characterization of a family of isostructural coordination polymers (CPs) with efficient luminescent properties. The CPs exhibit tailored crystal structures, efficient energy transfer to lanthanide ions, and intense emissions in the visible and near-infrared regions. Specifically, one of the compounds, 4Tb, demonstrates high quantum yield and excellent sensing capabilities for detecting nitroaromatic-like explosives, with a high detection capacity, a low limit of detection, and selectivity among other molecules [14].

In the next study, the electronic properties of hexagonal graphene quantum rings (n-HGQRs) are predicted using thermally assisted occupation density functional theory (TAO-DFT). The results show that as the size of n-HGQRs increases, a transition occurs from nonradical to polyradical ground states, which is attributed to the localization of active TAO orbitals at the inner and outer edges of the rings [15].

Another study investigates the plastic deformation mechanisms of Ni/Al_2_O_3_ interface systems under high-strain-rate tensile loading using classical molecular dynamics simulations. The results reveal that the fracture behavior and dislocation nucleation and propagation mechanisms are dependent on the interface structure, with Type II interfaces exhibiting higher yield strength due to more stable Ni–O bonds. This study provides insights into the deformation mechanisms of Ni/Al_2_O_3_ interfaces under extreme conditions, aiding in the understanding of complex interface dislocation structures and their behavior [16].

Another study investigates the magnetic moments of ruthenium nanoparticles with a face-centered cubic (fcc) packing structure using density functional theory (DFT). The results reveal that the smallest nano-dots exhibit significant magnetic moments and high spin electronic structures, which are found to be the most stable, providing valuable insights into the magnetic properties of ruthenium nanostructures and their potential applications in catalysis and other fields [17].

Another study addresses the challenge of studying the transient exposed surfaces in material morphology and proposes a computational model combining density functional theory calculations and Wulff construction to predict the available morphologies of ZnO. The model successfully matches the experimental field-emission scanning electron microscopy (FE-SEM) images, offering insights into the morphological evolution and properties of materials and providing a practical tool for understanding and optimizing the efficiency of metal-oxide-based materials through morphology design [18].

A study that focuses on utilizing machine learning and deep potential molecular dynamics (DEEPMD) to create a reliable neural network potential (NNP) for amorphous silicon nitride (SiN_x_) is also published in this Special Issue. The NNP is successfully applied to the SiNx model, and simulations reveal that Si_3_N_4_, with a higher proportion of nitrogen to silicon, exhibits superior mechanical properties, such as a larger elastic modulus and yield stress, attributed to increased coordination numbers and radial distribution function. The nitrogen-to-silicon ratio significantly influences the micro-level structure and macro mechanical properties of SiN_x_ [19].

The impact of different nitro species adsorption on carbon-doped boron nitride nanoribbons (BNNRs) using density functional theory is also presented. The findings reveal that the adsorption caused a switch from magnetic to non-magnetic behavior in the system, and certain species could undergo dissociation. The study also highlights the preference for interaction on nanosurfaces with dopants replacing the B sublattice, presenting potential technological applications for these systems [20].

The final contribution [21] highlights the importance of understanding the surface reactivity of titania-based materials, particularly on the nanoscale, in various applications. This study focuses on using theoretical characterization and computational approaches to analyze the structural features responsible for the Raman spectra of pure, stoichiometric TiO_2_ materials, providing insights into the interpretation and application of Raman spectroscopy in characterizing different forms of titania systems. 

With this, we hope that readers interested in materials modelling will find in this Special Issue a good overview of the field. Furthermore, we hope that the included works will inspire the scientific community to further push the boundaries of these interesting topics.
